# Survival Outcome and Prognostic Factors for Pancreatic Acinar Cell Carcinoma: Retrospective Analysis from the German Cancer Registry Group

**DOI:** 10.3390/cancers13236121

**Published:** 2021-12-04

**Authors:** Ekaterina Petrova, Joachim Wellner, Anne K. Nording, Rüdiger Braun, Kim C. Honselmann, Louisa Bolm, Richard Hummel, Monika Klinkhammer-Schalke, Sylke Ruth Zeissig, Kees Kleihues van Tol, Sylvia Timme-Bronsert, Peter Bronsert, Sergey Zemskov, Tobias Keck, Ulrich Friedrich Wellner

**Affiliations:** 1 Department of Surgery, Campus Lübeck, University Hospital Schleswig-Holstein, Ratzeburger Allee 160, 23562 Lübeck, Germany; ek.petrova@yahoo.de (E.P.); joachim.wellner@web.de (J.W.); AnneKristin.Nording@uksh.de (A.K.N.); ruediger.braun@uksh.de (R.B.); KimChristin.Honselmann@uksh.de (K.C.H.); Louisa.Bolm@uksh.de (L.B.); richard.hummel@uksh.de (R.H.); ulrich.wellner@uksh.de (U.F.W.); 2 German Cancer Registry Group of the Society of German Tumor Centers—Network for Care, Quality and Research in Oncology (ADT), 14057 Berlin, Germany; Monika.Klinkhammer-Schalke@klinik.uni-regensburg.de (M.K.-S.); zeissig@krebsregister-rlp.de (S.R.Z.); kleihuesvantol@adt-netzwerk.de (K.K.v.T.); 3 Institute of Surgical Pathology, Comprehensive Cancer Center Freiburg, Medical Center-University of Freiburg, Faculty of Medicine, University of Freiburg, 79106 Freiburg, Germany; sylvia.timme@uniklinik-freiburg.de (S.T.-B.); peter.bronsert@uniklinik-freiburg.de (P.B.); 4 Department of General Surgery, Bogomolets National Medical University, 01601 Kiev, Ukraine; s.zemskov@nmu.ua

**Keywords:** pancreatic acinar cell carcinoma, pancreatic cancer, German Cancer Registry Group

## Abstract

**Simple Summary:**

Less than 1% of all pancreatic malignancies are acinar cell carcinomas. Based on data from the German Cancer Registry Group, we performed a comparative analysis of characteristics and prognostic factors of pancreatic acinar cell carcinoma and the most common type of pancreatic cancer—pancreatic ductal adenocarcinoma. Compared to pancreatic ductal adenocarcinoma, patients with pancreatic acinar cell carcinoma were younger at the time of diagnosis and the percentage of males was higher. The prognosis of patients with pancreatic acinar carcinoma was better than that of patients with pancreatic ductal adenocarcinoma. Surgical resection was the strongest positive prognostic factor for pancreatic acinar cell carcinoma. The study shows that pancreatic acinar cell carcinoma has features distinct from pancreatic ductal adenocarcinoma. Radical resection should be advocated, whenever feasible.

**Abstract:**

Background: Pancreatic acinar cell carcinoma (PACC) is a distinct type of pancreatic cancer with low prevalence. We aimed to analyze prognostic factors and survival outcome for PACC in comparison to pancreatic ductal adenocarcinoma (PDAC), based on data from the German Cancer Registry Group. Methods: Patients with PACC and PDAC were extracted from pooled data of the German clinical cancer registries (years 2000 to 2019). The distribution of demographic parameters, tumor stage and therapy modes were compared between PACC and PDAC. The Kaplan–Meier method and Cox regression analysis were used to delineate prognostic factors for PACC. Propensity score matching was used to compare survival between PACC and PDAC. Results: There were 233 (0.44%) patients with PACC out of 52,518 patients with pancreatic malignancy. Compared to PDAC, patients with PACC were younger (median age 66 versus 70, respectively, *p* < 0.001) and the percentage of males was higher (66.1% versus 53.3%, respectively, *p* < 0.001). More patients were resected with PACC than with PDAC (56.2% versus 38.9%, respectively, *p* < 0.001). The estimated overall median survival in PACC was 22 months (95% confidence interval 15 to 27), compared to 12 months (95% confidence interval 10 to 13) in the matched PDAC cohort (*p* < 0.001). Surgical resection was the strongest positive prognostic factor for PACC after adjusting for sex, age, and distant metastases (hazard ratio 0.34, 95% confidence interval 0.22 to 0.51, *p* < 0.001). There was no survival benefit for adjuvant therapy in PACC. Conclusions: PACC has overall better prognosis than PDAC. Surgical resection is the best therapeutic strategy for PACC and should be advocated even in advanced tumor stages.

## 1. Introduction

**Figure 1:**
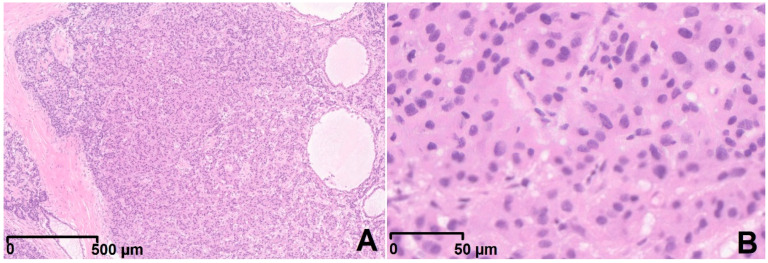
Overview of an acinar cell carcinoma with typical high cellularity and scant fibrous stroma (**A**, 2.5-fold magnification). In higher magnification (**B**, 20-fold magnification), tumor cells are presenting a granular eosinophilic cytoplasm and uniform nuclei (hematoxylin and eosin staining).

Pancreatic acinar cell carcinoma (PACC) is a distinct type of exocrine pancreatic cancer that arises from the acinic cells. It presents microscopically as a solid, cellular neoplasm with minimal stroma [1], (Figure 1). The prevalence of PACC has been estimated to be below 1% of all pancreatic malignancies [2]. Compared to patients with the most common pancreatic cancer, pancreatic ductal adenocarcinoma (PDAC), patients with PACC are on average younger and have a better overall prognosis [2,3]. Clinical evidence on PACC is based on case reports and small institutional series as well as a few registry-based studies that analyzed larger cohorts of patients with PACC [2,3,4,5,6,7]. Many of these studies point out surgical resection as the most effective therapeutic strategy [2,3,4,5,7]. The survival benefit of systemic therapy is more controversial [3,8,9].

Because of the low prevalence of PACC, clinical practices well studied in PDAC are often applied also to PACC. However, PACC has distinct clinical, histologic, and molecular features that make a differential approach necessary [1,2,4,10]. Especially relevant are the questions of long-term survival after resection of locally advanced tumors, resection of synchronous or metachronous distant metastases, as well as the benefit of different perioperative and palliative therapies. To expand our knowledge on a rare malignancy such as PACC, registry-based studies are the most feasible. 

In this study, we aimed to analyze prognostic factors and survival outcomes for PACC based on data from the German clinical cancer registries. To delineate the specifics of PACC, a comparative analysis to PDAC was performed.

## 2. Materials and Methods

### 2.1. Study Design and Ethics

This is a retrospective, registry-based study. It was approved by the local ethics committee of the University of Lübeck, Germany (Reference Number: 20-319).

### 2.2. German Clinical Cancer Registries

There is a longstanding tradition of hospital-based clinical cancer registration in Germany. Since 2013, clinical cancer registries have been established for quality assurance purposes in all federal states of Germany as part of the German National Cancer Plan. Therefore, in most of the federal states of Germany the preexisting epidemiological cancer registries have been expanded into population-based clinical registries [11]. The Society of German Tumor Centers (ADT) is the head organization that coordinates the German Cancer Registry Group on a national level. There is a uniform minimal data set for all oncological entities [12]. This includes items on demographics (age, sex), tumor entity, topography, and histology according to the International Classification of Diseases for Oncology (ICD-O) [13], TNM classification [14], therapy (operation, systemic therapy, radiation), and survival follow-up. The combined data from 17 clinical registries from the years 2000 to 2019 were used according to the data use rules of the German Cancer Registry Group. 

### 2.3. Patients

Of all patients with pancreatic malignancy (codes C25.0-C25.9, ICD-O 3. edition (ICD-O-3)), patients with PACC (ICD-O-3 morphology code 8550/3) and not otherwise specified PDAC (ICD-O-3 morphology code 8500/3) were extracted. Patients with other pancreatic neoplasms with acinar differentiation [1], such as pancreatoblastoma (ICD-O-3 morphology code 8971/3) or mixed ductal-acinar carcinoma (ICD-O-3 morphology code 8552/3), were not the subject of this study.

### 2.4. Study Parameters

The following parameters were selected: histological diagnosis (PACC, PDAC), age at diagnosis (years), therapy (none, operation alone, neoadjuvant chemotherapy/radiochemotherapy plus operation with/without adjuvant therapy, operation plus adjuvant chemotherapy/radiochemotherapy, chemotherapy/radiochemotherapy alone), operation type (pancreatoduodenectomy, distal pancreatectomy, total pancreatectomy, other), type of chemotherapy (gemcitabine mono, 5-FU mono, platin-based, other), distant metastases (M0, M1, Mx), lymph node metastases (N0, N1, Nx), T-stage (T0–T4), lymph vessel invasion (L0, L1), vascular invasion (V0, V1), grading (G1-G4), resection status (R0, R1, R2), follow-up (months after diagnosis), status at last follow-up (dead, alive). The variables age, grading, and resection status were dichotomized as follows: age in ≤67 years versus >67 years, grading in G1/2 versus G3/4, resection status in R0 versus R1/R2 (R+). Distant metastases were considered positive M1, whenever either pathologic pM1 or clinical cM1 were given. T-stage and N-stage were evaluated based on the pathological stages pT and pN. Since the TNM classification changed over the selected period (2000–2019), we made no differentiation between N1 and N2. Additionally, stage T2 and T3 were combined, since restaging was not possible without data on tumor size [15].

### 2.5. Statistical Methods

Differences in the overall distribution of the various parameters between PACC and PDAC were delineated. Additionally, multivariable analysis with covariables age, sex, distant metastases, and operation was performed. With the subgroup of patients with upfront surgery (resection without prior neoadjuvant therapy), univariable survival analysis with covariables age, sex, T-stage, lymph node metastases, distant metastases, vessel invasion, lymph vessel invasion, resection status, adjuvant therapy was performed. Data processing and statistical analysis were performed with R version 3.3.3 [16]. Descriptive statistics with median and interquartile range of continuous variables and absolute number and percentage of total for categorical variables were used. Univariable logistic regression was applied in the comparison of the distribution of the parameters studied between PACC and PDAC. The propensity score was calculated via logistic regression with covariables age, sex, therapy, T-stage, lymph node metastases, distant metastases, resection status, grading, year of diagnosis, survival status. Propensity score nearest neighbor matching was performed. The R package MatchIt was used [17].

### 2.6. Survival Analysis

For patients with available survival data, propensity score matching of PACC to PDAC in ratio 1:3 was performed. Survival analysis for PACC and PDAC was performed with the matched data. Univariable survival analysis for age, sex, distant metastases, grading, and therapy type was applied to the whole data set. Kaplan–Meier method and log-rank test were applied in the univariable survival analysis. Additionally, multivariable Cox regression with covariables age, sex, distant metastases, and operation was performed. With the subgroup of patients with upfront surgery (resection without prior neoadjuvant therapy), univariable survival analysis with covariables age, sex, T-stage, lymph node metastases, distant metastases, vessel invasion, lymph vessel invasion, resection status, adjuvant therapy was performed. Follow-up was estimated with the reversed Kaplan–Meier method. Significance level 0.05 and confidence interval 95% were set. 

## 3. Results

### 3.1. Basic Characteristics of the PACC Cohort

There were 233 (0.44%) patients with PACC out of 52,518 patients with pancreatic malignancy in the whole dataset. The median age at time of diagnosis was 66 years. There were 154 (66.1%) male and 79 (33.9%) female patients. Distant metastases were present in 79 (33.9%) patients. Resection was performed in 131 (56.2%) cases. Of those resected, 80 (61%) received operation alone, 49 (37.4%) operation and adjuvant therapy (47 chemotherapy, 2 radiochemotherapy), 2 (1.5%) neoadjuvant chemotherapy and operation (one of them received neoadjuvant and adjuvant chemotherapy). Distant metastases M1 were present in 18 (13.7%), and lymph node metastases N1 in 56 (42.7%) of resected patients. Results of the univariable analysis are given in Table 1. 

### 3.2. Survival Analysis of PACC Patients

**Figure 2:**
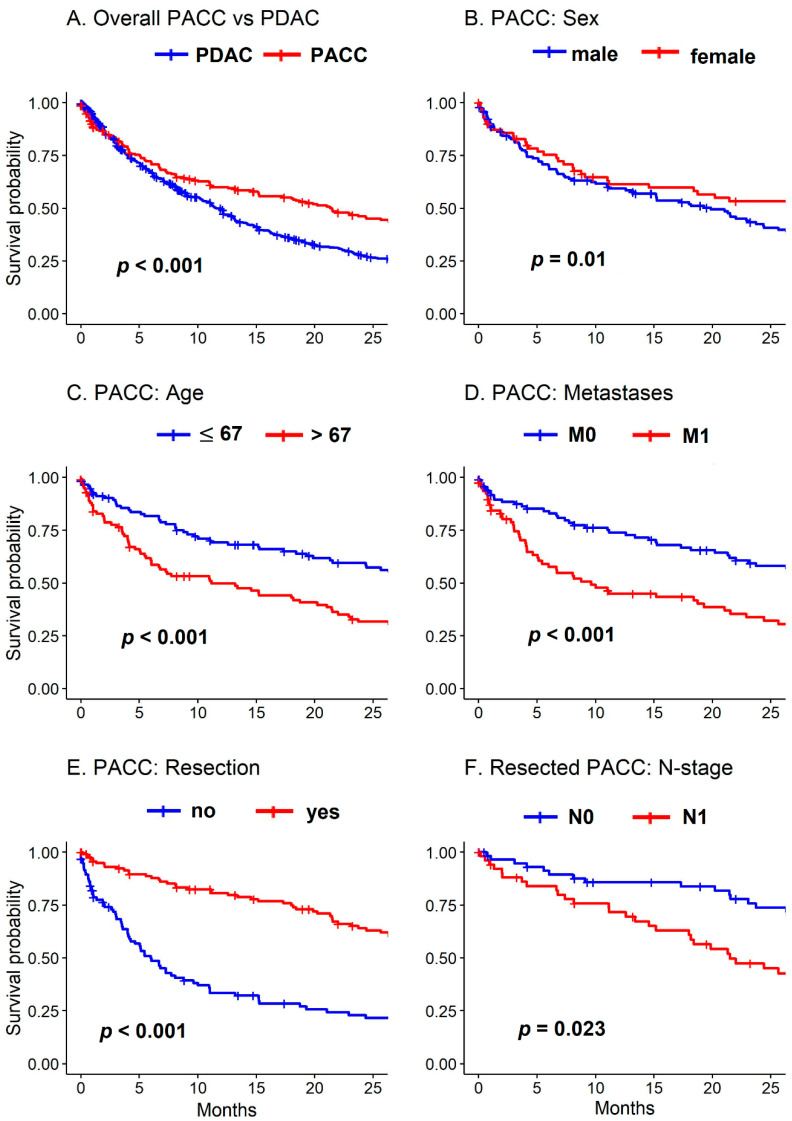
Survival of PACC and matched PDAC cohorts. (**A**) Overall survival of PACC versus PDAC. (**B**) Survival of PACC patients according to sex. (**C**) Survival of PACC patients according to age (67 years or younger versus older than 67 years). (**D**) Survival of PACC patients according to distant metastases. (**E**) Survival of PACC patients according to resection versus no resection. (**F**) Survival of PACC patients according to lymph node metastases.

Survival data were available for 218 (93.6%) patients. Of those, 157 (72%) were dead at last follow-up. Median follow-up was 92 (56 to 158) months. Median overall survival was 22 (15 to 27) months (Figure 2A), 5-year overall survival rate was 21.5% (16.1 to 28.8%). Median survival of resected patients was 34 (27 to 45) months, 5-year survival rate 33.1% (24.8 to 44.3%). Non-resected patients had a median overall survival of 6 (4 to 10) months. (Figure 2E)

In the univariable survival analysis, females had better overall prognosis than males (31 versus 20 months, respectively, *p* = 0.01) (Figure 2B). Age > 67 years (11 versus 29 months, *p* < 0.001) (Figure 2C) as well as distant metastases (10 versus 28 months, *p* < 0.001) were negative prognostic factors (Figure 2D). The results of the univariable survival analysis are given in Table 2. 

In the multivariable Cox regression, female sex (hazard ratio (HR) 0.6 (0.43 to 0.87), *p* = 0.006) and resection were positive prognostic factors (HR 0.34 (0.22 to 0.51), *p* < 0.001), while age > 67 was a negative prognostic factor (HR 2.1 (1.51 to 2.91), *p* < 0.001) (Table 3).

In the subgroup of patients with upfront surgery, lymph node metastases (22 versus 45 months, *p* = 0.023) (Figure 2F) and lymph vessel invasion (21 versus 45 months, *p* = 0.003) were associated with worse prognosis in the univariable survival analysis. 

### 3.3. Comparison PACC and PDAC

PACC patients were compared to a cohort of 37,940 patients with PDAC (72.2% of all 52,518 patients). The median age at time of diagnosis of PDAC patients was 70 years compared to 66 years of PACC patients (*p* < 0.001). The proportion of male patients was higher in the PACC cohort (66.1% versus 53.3%, *p* < 0.001). More patients were resected in the PACC cohort (56.2% versus 38.9%, *p* < 0.001). Of those resected, more patients received adjuvant chemotherapy in the PACC cohort (38.2% versus 30.1%, *p* = 0.045). PACC patients underwent distal pancreatectomy more often than PDAC patients (29 versus 8.2%, respectively, *p* < 0.001) (Table 1). The estimated overall survival after diagnosis for PACC patients was 22 (15 to 27) months, and for all PDAC patients, this was 8.1 (8.07 to 8.13) months (*p* < 0.001). 

The 218 PACC patients with available survival data were matched to 654 patients with PDAC. The estimated overall survival of the matched PDAC cohort was 12 (10 to 13) months (Figure 2A). Females had better prognosis in PACC (31 versus 20 months, respectively, *p* = 0.01) compared to males but not in PDAC (13 versus 11 months, respectively, *p* = 0.163). There were several differences between PACC and the matched PDAC in the subgroup of patients with upfront surgery: there was no statistically significant difference in the estimated survival of resected patients without (M0) and with distant metastases (M1) in PACC (29 versus 32 months, respectively, *p* = 0.709) but significantly shorter survival for M1 in PDAC (24 versus 9 months, respectively, *p* < 0.001); no significant difference in resection margin status in PACC R0 versus R+ (34 versus 23 months, respectively, *p* = 0.208) but significant difference in PDAC (29 versus 12 months, respectively, *p* < 0.001); no significant difference with adjuvant therapy versus no adjuvant therapy in PACC (36 versus 34 months, respectively, *p* = 0.48) but survival benefit for PDAC patients with adjuvant therapy (30 versus 16 months, respectively, *p* < 0.001).

## 4. Discussion

The results of the current analysis are in line with the results of previous studies, showing that PACC is a rare type of pancreatic cancer, occurring more often in males and at a younger age, compared to PDAC. Estimated overall median survival was better in PACC compared to a matched PDAC cohort (22 versus 12 months, respectively, *p* < 0.001). Resection, younger age, and female sex were independent positive prognostic factors. No survival benefit could be shown for systemic therapy.

Most of the large-scale studies on PACC are based on data from the American National Cancer Database (NCDB) as well as from the Surveillance, Epidemiology, and End Results (SEER) registry [3,5,8,18,19,20]. Some of these studies analyze overlapping cohorts, focusing on different aspects [5,8,18,19,20]. Table 4 gives an overview of publications with more than 50 patients included. Three of them, Wisnoski et al., Schmidt et al., and Huang et al., presented a comparative analysis of PACC to PDAC [2,3,21].

Demographic characteristics of the European cohort described in this study were comparable to those reported of cohorts from the United States. Most of the NCBI/SEER-based studies reported a median age of PACC patients about 65 years. Patients in the two Japanese publications, Kitagami et al. and Takahashi et al., were younger with median ages 60 and 61 years [9,22]. In the current study, the median age for PACC patients was 66 years, compared to 70 years for PDAC patients (*p* < 0.001). Similarly, a higher median age for PDAC was reported by Wisnoski et al. (56 vs. 70 years, *p* < 0.001) and Schmidt et al. (67 vs. 70 years, *p* < 0.001) [2,3]. Huang et al. reported a higher mean age for PDAC (50.8 vs. 59.4 years, *p* > 0.001) [21].

All but one study reported the predominance of the male sex with 54–74%. In our study, the proportion of males in PACC was 66.1% and, thus, significantly higher than in PDAC (53.3%, *p* < 0.001). A significantly higher male proportion in PACC than in PDAC was reported also in Wisnoski et al. (53.6 vs. 48.4%, respectively, *p* < 0.01) and Schmidt et al. (63.5 vs. 49.9%, respectively, *p* < 0.001) [2,3]. 

The proportion of PACC patients with distant metastases was lower than that of PDAC patients (33.9% vs. 40%, respectively, *p* = 0.002). However, because of the high proportion of unknown metastases status Mx in both groups (20.6% in PACC versus 25.8% in PDAC), the actual distribution might be different. In resected patients, lymph node metastases N1 were more common in PDAC (63.7%) than in PACC (42.7%, *p* < 0.001). Here, the proportion of unknown lymph node status Nx was much lower (4.6% PACC and 7.8% PDAC) and, thus, would not impact the N0 to N1 distribution significantly. Schmidt et al. similarly reported a higher proportion of lymph node metastases in PDAC (N1 PACC 32.1% versus 48.5% PDAC) [2]. Wisnoski et al. reported higher proportions of distant metastases M1 and lymph node metastases N1 for PACC, but again here the proportion of Mx and Nx was too high to draw a conclusion about the actual distribution [3].

PACC showed a much better prognosis with an estimated median survival time of 22 months (95% CI 15 to 27) compared to the PDAC cohort with 8.1 months (95% CI 8.07 to 8.13). To account for the differences in stage, sex, and age distribution as well as differences in therapies applied, we performed a propensity score matching of 1:3 PACC to PDAC patients. After matching, the difference in overall median survival was smaller but still significantly better in PACC (22 vs. 12 months, *p* < 0.001). Resection was the strongest positive prognostic factor for PACC, after adjustment for age, sex, and distant metastases (OR 0.34, 95% CI 0.22 to 0.51, *p* < 0.001). 

Among resected PACC patients, lymph node metastases were a negative prognostic factor. Positive resection margin was not associated with survival, but the analysis might be biased and underpowered because of missing data. It is noteworthy that distant metastases were a negative prognostic factor in resected PDAC patients but not in PACC patients. Though this result should be interpreted with caution due to potential bias, metastases resection should be given consideration for PACC patients. Similar to our study, Landa et al. reported 12% of 566 resected PACC patients to be stage IV, with an estimated 5-year survival of 19% versus 4% (*p* < 0.001) of not resected stage IV patients [5]. There have been several case reports of successful curative resections of metastases in PACC [24,25,26,27,28,29]. Ohara et al. reported the successful resection of metachronous rectal and liver metastases, Kittaki et al. and Villano et al. performed resections of liver metastases after systemic therapy, and Suzuki et al. published a case of a patient with long-term survival after repetitive resection of recurrent liver metastases [26,27,28,29]. Hartwig et al. reported the successful resection in four out of six patients with metastatic PACC [24]. Besides resection, radiofrequency ablation has been reported to be successful in treating liver metastases in PACC patients and should be given consideration in the multimodal treatment strategy [30,31]. 

Our study did not demonstrate any survival benefit of systemic therapy in PACC, while adjuvant as well as palliative systemic therapy was associated with better prognosis in the matched PDAC cohort. In a recent study, Patel et al. demonstrated a survival benefit for adjuvant chemotherapy in PACC in a multivariable Cox regression analysis [8]. Because of the small number of patients (*N* = 131) and differently distributed missing data among the variables, we did not perform a multivariable analysis for the subgroup of resected patients. Furthermore, the most common regime of adjuvant chemotherapy given for PACC was gemcitabine monotherapy in 30 patients (60% of patients with adjuvant chemotherapy). The results of several previous studies suggest that gemcitabine monotherapy might not lead to a response in PACC patients but, rather, platin-based regimes should be given preference [4,9,23,32,33,34]. Thus, the lack of overall survival benefit of adjuvant therapy in our study might also be due to the high proportion of patients with gemcitabine monotherapy. 

Studies of the molecular biology of PACC have shown frequent aberrant DNA methylation and chromosomal abnormalities, and it has been reported that about one third of PACC have targetable genetic alterations in genes such as BRAF, BRCA2, SMAD4, RAF1, NTRK1 [10,35]. Corresponding targeted therapies have been successfully applied [32,36,37]. Thus, molecular profiling should have an integral role in guiding therapeutic decisions in patients with PACC.

## 5. Conclusions

It is important to bear in mind that PACC has features distinct from PDAC. Radical surgical resection should be advocated, including resection of metastases, whenever feasible. Though the role of systemic therapy is not well defined, it should be considered at least for patients with advanced disease.

## Figures and Tables

**Table 1:** Univariable analysis PACC versus PDAC.

Parameter	*N* (% of Total)/Median (IQR)		*p*-Value
	PACC	PDAC	
**All Patients**			
Total number	233 (100)	37,940 (100)	
Sex			
male	154 (66.1)	20,225 (53.3)	
female	79 (33.9)	17,712 (46.7)	<0.001
missing	0 (0)	3 (<0.001)	0.978
Median age [years]	66 (17)	70 (14)	<0.001
Age			
≤67 years	128 (54.9)	15,863 (41.8)	
>67 years	105 (45.1)	22,077 (58.2)	<0.001
Distant metastases			
M0	106 (45.5)	12,964 (34.2)	
M1	79 (33.9)	15,177 (40)	0.002
Mx	48 (20.6)	9799 (25.8)	0.003
Treatment			
none	59 (25.3)	15,249 (40.2)	
Operation alone	80 (34.3)	10,139 (26.7)	<0.001
neoadjuvant + operation	2 (0.9)	299 (0.8)	0.448
operation + adjuvant	49 (21)	4304 (11.3)	<0.001
(Radio-)chemotherapy	43 (18.5)	7949 (21)	0.095
Operation	131 (56.2)	14,742 (38.9)	<0.001
**Subgroup of resected patients**			
Total number	131 (100)	14,742 (100)	
Operation type			
PD	36 (27.5)	6303 (42.8)	
DPR	38 (29)	1216 (8.2)	<0.001
TPE	8 (6.1)	903 (6.1)	0.263
Other/not specified	49 (37.4)	6320 (42.9)	0.165
T-stage			
pT0	1 (0.8)	23 (0.2)	
pT1	6 (4.6)	501 (3.4)	0.242
pT2/3	111 (84.7)	12,323 (83.6)	0.125
pT4	8 (6.1)	1367 (9.3)	0.064
pTx	5 (3.8)	528 (3.6)	0.172
Lymph node metastases			
N0	69 (52.7)	4208 (28.5)	
N1	56 (42.7)	9385 (63.7)	<0.001
Nx	6 (4.6)	1149 (7.8)	0.007
Distant metastases			
M0	97 (74)	10,240 (69.5)	
M1	18 (13.7)	2465 (16.7)	0.312
Mx	16 (12.2)	2037 (13.8)	0.489
Resection margin status			
R0	69 (52.7)	6280 (42.6)	
R1	15 (11.5)	2481 (16.8)	0.037
R2	9 (6.9)	750 (5.1)	0.805
missing	38 (29)	5231 (35.5)	0.041
Grading			
G1	15 (11.5)	569 (3.9)	
G2	31 (23.7)	6735 (45.7)	<0.001
G3	36 (27.5)	5897 (40)	<0.001
G4	2 (1.5)	80 (0.5)	0.945
missing	47 (35.9)	1461 (9.9)	0.508
Lymph vessel invasion			
L0	39 (29.8)	3433 (23.3)	
L1	43 (32.8)	5158 (35)	0.164
missing	49 (37.4)	6151 (41.7)	0.1
Blood vessel invasion			
V0	53 (40.5)	6213 (42.1)	
V1	26 (19.8)	2088 (14.2)	0.116
missing	52 (39.7)	6441 (43.7)	0.779
Adjuvant therapy	50 (38.2)	4432 (30.1)	0.045
Adjuvant chemotherapy type			
gemcitabine mono	30 (22.9)	2919 (19.8)	
5-FU mono	2 (1.5)	124 (0.8)	0.54
platin-based	9 (6.9)	363 (2.5)	0.022
other	9 (6.9)	1026 (7)	0.678
none	81 (61.8)	10,310 (69.9)	0.211

N—number; DPR—distal pancreatectomy; PACC—pancreatic acinar cell carcinoma; PD—pancreatoduodenectomy; PDAC—pancreatic ductal adenocarcinoma; TPE—total pancreatectomy.

**Table 2:** Univariable survival analysis for PACC and the propensity score matched PDAC cohort.

	PACC				PDAC			
Parameter	*N*	Deaths	Median Survival in Months (95% CI)	*p*-Value	*N*	Deaths	Median Survival in Months (95% CI)	*p*-Value
**All Patients**								
Overall	218	157	22 (15 to 27)		654	498	12 (10 to 13)	
Sex								
male	145	108	20 (13 to 26)	0.01	443	343	11 (9 to 12)	0.163
female	73	49	31 (15 to 67)		211	155	13 (11 to 16)	
Age								
≤67 years	118	73	29 (24 to 52)	<0.001	354	242	15 (12 to 18)	<0.001
>67 years	100	84	11 (6 to 21)		300	256	8 (7 to 11)	
Distant metastases								
M0	99	63	28 (23 to 42)	<0.001	302	204	23 (19 to 28)	<0.001
M1	78	65	10 (5 to 22)		238	208	6 (5 to 7)	
Therapy								
none	52	42	5 (3 to 11)	<0.001	156	126	4 (3 to 6)	<0.001
Operation alone	72	49	34 (24 to 45)		216	172	16 (13 to 19)	
neoadjuvant + operation	2	1	NA		6	4	NA	
operation + adjuvant	49	27	36 (26 to 69)		147	82	30 (24 to 43)	
Cx/Rcx	43	38	6 (4 to 15)		129	114	7 (6 to 9)	
Operation								
no	95	80	6 (4 to 10)	<0.001	285	240	5 (4 to 6)	<0.001
yes	123	77	34 (27 to 45)		369	258	20 (18 to 24)	
**Subgroup of resected patients without neoadjuvant treatment**								
Overall	123	77	34 (27 to 45)		369	258	20 (18 to 24)	
Sex								
male	77	53	27 (24 to 40)	0.004	242	176	19 (15 to 23)	0.169
female	44	23			121	78	22 (18 to 30)	
Age								
≤67 years	69	38	53 (36 to 152)	0.001	209	135	23 (20 to 30)	0.003
>67 years	52	38	24 (20 to 34)		154	119	16 (13 to 22)	
T-stage								
pT1	6	2	NA		19	14	52 (15 to NA)	<0.001
pT2/3	103	64	34 (26 to 42)		315	216	22 (18 to 25)	
pT4	7	7	15 (2 to NA)		16	16	7 (5 to 17)	
Lymph node metastases								
N0	63	35	45 (29 to 99)	0.023	190	116	34 (24 to 48)	<0.001
N1	52	38	22 (18 to 36)		159	125	15 (12 to 18)	
Distant metastases								
M0	89	55	29 (26 to 45)	0.709	266	178	24 (22 to 32)	<0.001
M1	16	13	32 (22 to 69)		61	53	9 (6 to 13)	
Lymph vessel invasion								
L0	36	18	45 (30 to NA)	0.003	116	66	30 (24 to 48)	0.003
L1	42	32	21 (15 to 32)		114	80	22 (18 to 26)	
Blood vessel invasion								
V0	50	29	29 (23 to 56)	0.107	171	99	30 (23 to 47)	<0.001
V1	25	17	18 (15 to 42)		48	38	16 (9 to 22)	
Resection margin status								
R0	66	34	34 (26 to 99)	0.208	207	121	29 (23 to 40)	<0.001
R+	23	18	23 (15 to 69)		51	44	12 (9 to 25)	
Grading								
G1/G2	42	33	29 (23 to 45)	0.262	140	108	24 (20 to 34)	0.008
G3/G4	35	25	18 (11 to 32)		103	77	15 (10 to 19)	
Adjuvant Therapy								
no	72	49	34 (24 to 45)	0.48	216	172	16 (13 to 19)	<0.001
yes	49	27	36 (26 to 69)		147	82	30 (24 to 43)	

CI—confidence interval; Cx- chemotherapy; N—number; NA—not available; PACC—pancreatic acinar cell carcinoma; PDAC—pancreatic ductal adenocarcinoma; Rcx—radiochemotherapy.

**Table 3:** Multivariable Cox regression for PACC patients with available survival data (*N* = 218).

Parameter	HR (95% CI)	*p*-Value
Sex (male vs. female)	0.6 (0.43 to 0.87)	0.006
Age (≤67 vs. >67 years)	2.1 (1.51 to 2.91)	<0.001
Distant metastases (M1 vs. M0)	1.2 (0.78 to 1.87)	0.408
Distant metastases (Mx vs. M0)	0.71 (0.43 to 1.16)	0.173
Resection	0.34 (0.22 to 0.51)	<0.001

CI—confidence interval; HR—hazard ratio; PACC—pancreatic acinar cell carcinoma.

**Table 4:** Summary of studies on PACC with more than 50 patients included.

First Author	Year of Publication	Data Source	Years of Diagnosis	*N*	Median Age	Male %	Median Survival in Months (Range)	5-Year Survival Rate (%)
Kitagami [22]	2007	JPS registry	1981 to 2004	115	60	66.9	nr	nr
Wisnoski [3]	2008	SEER	1988 to 2003	672	56	53.6	47 (nr)	42.8
Schmidt [2]	2008	NCDB	1985 to 2005	865	67	63.5	nr	nr
He [18]	2018	SEER	2004 to 2014	227	65	25.6	16 (11–20)	nr
Landa [5]	2018	NCDB	1998 to 2012	980	64	68	nr	nr
Patel [8]	2020	NCDB	2004 to 2015	298	nr	72.8	60 (nr)	nr
Duorui [20]	2020	SEER	2004 to 2016	252	64	70.6	nr	17.5
Zong [19]	2020	SEER + institu-tional	2005 to 2015 + 2005 to 2018	306	67	73.5	27 (19–35)	36.8
Takahashi [9]	2021	Multi-center	1996 to 2013	58	61	69	13 (8–19)	nr
Huang [21]	2021	Single-center	2006 to 2016	52	nr	59.6	39 (nr)	21.4
Sridharan [23]	2021	Multi-center	1996 to 2019	66	64	70	24.7	

CI—confidence interval; JPS—Japan Pancreas Society; N—number; NCDB—National Cancer Database, nr—not reported; PACC—pancreatic acinar cell carcinoma; SEER—Surveillance, Epidemiology, and End Results.

## Data Availability

Data were obtained from The Society of German Tumor Centers (Arbeitsgemeinschaft Deutscher Tumorzentren, ADT) and are available from the authors with the permission of The Society of German Tumor Centers.
